# Ultrasound-Driven Conformational Rearrangement of *Coilia ectenes taihuensis* Myofibrillar Protein Enhances Emulsion Stability

**DOI:** 10.3390/molecules31213966

**Published:** 2026-11-03

**Authors:** Xinhui Zhou, Zhangyan Sun, Lijun Zhu, Suting Zhuang, Xiu Wang, Changjun Zhu, Mingfeng Xu

**Affiliations:** 1 Zhejiang Provincial Modern Biology and Medicine Industry College, College of Life and Environmental Sciences, Hangzhou Normal University, Hangzhou 311121, China; 2025112010036@stu.hznu.edu.cn (X.Z.); 2025112010057@stu.hznu.edu.cn (Z.S.); 2026112010078@stu.hznu.edu.cn (L.Z.); 2026112010017@stu.hznu.edu.cn (S.Z.); 2 School of Advanced Materials Engineering, Jiaxing Nanhu University, Jiaxing 314001, China; 3 College of Biological and Chemical Engineering, Jiaxing University, Jiaxing 314001, China; zhuchangjun@mail.zjxu.edu.cn

**Keywords:** *Coilia ectenes taihuensis*, myofibrillar protein, ultrasound treatment, conformational rearrangement, emulsion stability

## Abstract

Myofibrillar protein (MP) from *Coilia ectenes taihuensis* (CMP) has potential as a natural emulsifier; however, its relatively compact native structure may restrict its functional performance. This study evaluated the effects of ultrasound treatment (0–600 W) on CMP conformational characteristics and the stability of CMP-based emulsions. Ultrasound-induced power-dependent structural and oxidative modifications in CMP, as evidenced by increased carbonyl content, decreased sulfhydryl content, secondary structure rearrangement, and altered surface hydrophobicity. Under the conditions tested, CMP treated at 450 W exhibited the highest surface hydrophobicity and enhanced solubility, which were associated with improved emulsifying properties. The emulsifying activity index increased from 39.93 m^2^/g to 82.17 m^2^/g, accompanied by reduced droplet size, higher absolute ζ-potential values, enhanced viscoelastic properties, and a reduced creaming index. However, at 600 W, further structural modification and possible protein association may have contributed to the decline in emulsifying performance. These findings suggest that appropriate ultrasound treatment can improve the emulsifying performance of CMP under the conditions investigated, providing insights into its potential application in protein-based emulsion systems.

## 1. Introduction

Protein-stabilized emulsions are increasingly explored for applications in functional food products, nutrient delivery systems, and encapsulation technologies [1]. Their stability largely depends on the physicochemical and structural attributes of the proteins involved, which in turn influence protein functionality during emulsion formation and stabilization. Compared with conventional low-molecular-weight emulsifiers, naturally derived proteins possess advantages such as biocompatibility, nutritional value, and environmental sustainability, making them promising candidates for developing functional emulsion systems [1].

*Coilia ectenes taihuensis* (*C. ectenes taihuensis*) is an economically valuable freshwater fish distributed in the Taihu Lake Basin, China [2]. In fish muscle, myofibrillar proteins (MPs) constitute the predominant protein fraction and are primarily composed of myosin and actin, representing approximately 55–65% of total muscle protein [3,4]. Due to their amphiphilic characteristics, MPs possess intrinsic emulsifying potential and have been widely investigated as natural protein-based emulsifiers in emulsion systems [5]. However, the compact and highly organized native structure of MPs may restrict conformational flexibility and limit their functional performance in emulsion systems [4]. Accordingly, different modification strategies have been explored to regulate the structural and functional properties of muscle proteins and improve their performance in food systems [6,7]. Compared with commonly studied fish species, the structural characteristics and functional regulation of MPs from *C. ectenes taihuensis* remain largely unexplored. Therefore, developing effective physical modification strategies to regulate the conformation and functionality of CMP is important for improving the functional properties of freshwater fish proteins in emulsion systems.

Non-thermal processing technologies, such as ultrasound and cold plasma, have been increasingly applied in protein modification and emulsion preparation because of their simple operation and high processing efficiency [8,9,10]. Acoustic cavitation can modify the structural properties of MPs through the disruption of hydrophobic forces and hydrogen bonding, thus causing conformational unfolding and structural rearrangement [11]. Ultrasound treatment also promoted a more flexible MP conformation and increased the accessibility of hydrophobic domains, which contributed to better emulsifying performance and emulsion stability [12]. Wang et al. [13] also linked sonication to improved viscoelastic behavior and storage stability in protein-based emulsions, indicating that ultrasound-induced structural modification may contribute to enhanced emulsion stability.

Ultrasound-induced structural modification and functional regulation of MPs have been investigated in protein systems from different animal species. The study by Zhou et al. [14] demonstrated that Antarctic krill myofibrillar proteins exhibited enhanced gelling and emulsifying properties after ultrasound treatment. Similarly, ultrasound-driven conformational modulation of chicken myofibrillar proteins was shown to alter protein structural characteristics and enhance their functional properties [15]. Moreover, high-intensity ultrasound modification of silver carp myofibrillar proteins was reported to regulate protein structural characteristics and enhance the stability of high internal phase emulsion systems [16]. Ultrasound-induced structural remodeling has also been investigated in oleosome-derived proteins in relation to emulsion stability [17]. However, previous studies have primarily demonstrated that ultrasound treatment can modify the structure and functional properties of myofibrillar proteins, whereas how progressive changes in ultrasound power translate into specific structural modifications and subsequent changes in emulsifying and emulsion-stabilizing properties remains insufficiently understood. In particular, the structure–function relationship across different ultrasound intensities has not been systematically established, making it difficult to distinguish structural changes that are associated with improved interfacial functionality from those that may become unfavorable at higher treatment intensities. In addition, the ultrasound-responsive characteristics and emulsion-stabilizing behavior of myofibrillar protein from *C. ectenes taihuensis*, a commercially important freshwater fish of the Taihu Lake Basin, remain unexplored.

We hypothesized that ultrasound power would differentially modulate the conformation and surface characteristics of CMP, which would in turn alter its emulsifying efficiency and ability to stabilize oil-in-water emulsions. Accordingly, the present work was designed to investigate the power-dependent changes in the structural characteristics and emulsion-stabilizing behavior of myofibrillar protein from *C. ectenes taihuensis*. To achieve this, we systematically varied the ultrasound power over a gradient (0–600 W) and integrated analyses of protein conformation, emulsifying properties, droplet characteristics, ζ-potential, microstructure, rheological properties, and storage stability. This study aimed to elucidate the association between ultrasound-induced structural modification and the interfacial and stability-related properties of CMP, with the broader goal of providing guidance for developing freshwater fish proteins as functional ingredients in emulsion-based food systems.

## 2. Results and Discussion

### 2.1. Total Sulfhydryl Content

**Figure 1:**
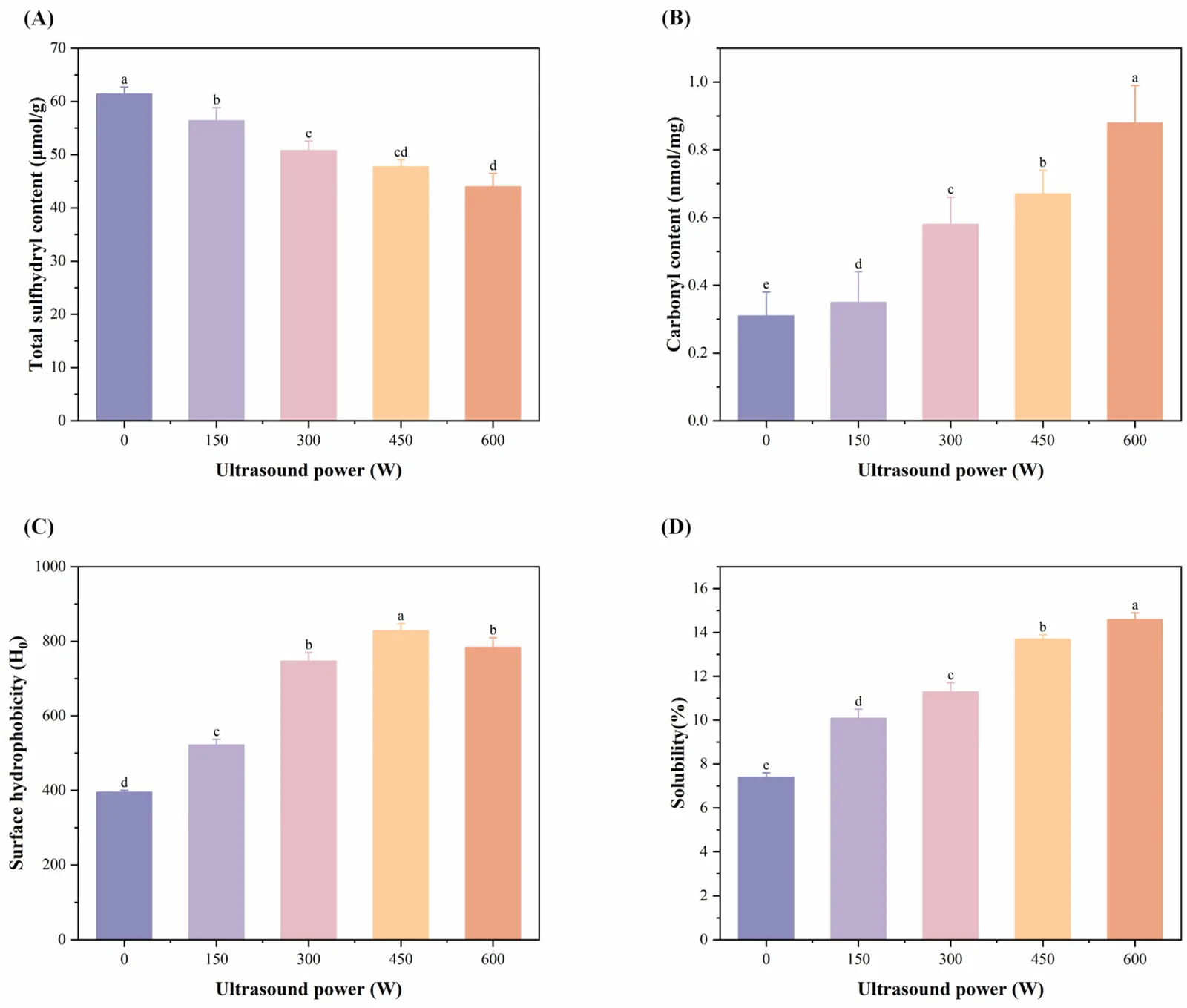
Effects of ultrasound power on total sulfhydryl content (**A**), carbonyl content (**B**), surface hydrophobicity (**C**), and solubility (**D**) of CMP. Different lowercase letters indicate significant differences between treatments (*p* < 0.05).

The quantification of total sulfhydryl groups is routinely used to monitor protein conformational alterations and the extent of cross-linking. Changes in sulfhydryl content induced by ultrasound can markedly influence the functional performance of MP [18]. The ultrasound-treated CMP exhibited a marked decrease in total sulfhydryl content compared with the untreated CMP (Figure 1A). Moreover, a progressive reduction was observed with increasing ultrasound power, with the total sulfhydryl content decreasing from 61.4 to 44.0 nmol/mg under the 600 W ultrasound treatment. The lower sulfhydryl content could be partly associated with oxidative modification of thiol groups, potentially involving reactive species such as hydroxyl radicals (·OH) generated during acoustic cavitation [19]. Meanwhile, ultrasound-generated shear forces may loosen the protein conformation, increasing the accessibility of buried sulfhydryl groups and their susceptibility to oxidative reactions. The observed decrease in total sulfhydryl content may thus reflect changes in the chemical state or accessibility of thiol groups following ultrasound treatment, which may be associated with alterations in protein structure and functional properties.

### 2.2. Carbonyl Content

The level of protein carbonyls is commonly used as a marker of oxidative changes in proteins. As presented in Figure 1B, carbonyl content in CMP increased significantly with increasing ultrasound power, suggesting an increase in oxidation-related modification following ultrasonic treatment. This increase may be associated with reactive species generated during acoustic cavitation, which could react with oxidation-sensitive amino acid side chains and contribute to carbonyl formation. Zhao et al. [20] further reported that free radicals generated during ultrasound treatment oxidatively modified amino acid residues and accelerated carbonyl formation, leading to alterations in protein conformation and physicochemical characteristics. Abdelnaby et al. [21] also suggested that side chains containing amino or imino groups, such as -NH_2_ and -NH, may be oxidatively transformed into carbonyl compounds. These findings suggest that ultrasound treatment altered the oxidation-related characteristics of CMP, which may influence its structural properties and emulsifying performance. Therefore, the relationship between ultrasound-induced structural/oxidation-related changes and protein functional improvement requires further investigation.

### 2.3. Surface Hydrophobicity

The surface hydrophobicity index provides information on the exposure of hydrophobic regions and changes in protein conformation. After ultrasound treatment, CMP exhibited a marked increase in this index, reaching its maximum value of 828.70 at 450 W (Figure 1C). This finding suggests that ultrasound treatment may have altered the protein conformation and increased the exposure of hydrophobic groups [22]. At 600 W, however, surface hydrophobicity decreased to 784.27. This decrease may be associated with further intermolecular interactions that partially reduced the accessibility of hydrophobic groups. Huang et al. [23] similarly reported that moderate ultrasound treatment facilitated hydrophobic group exposure, whereas excessive treatment induced structural rearrangement and a slight reduction in H_0_. The increased hydrophobic exposure under moderate sonication may enhance CMP emulsifying performance. A similar dose-dependent rearrangement has been reported for quercetin-modified silkworm pupa protein [24].

### 2.4. Solubility

Protein solubility is influenced by the degree of structural modification and intermolecular association and is closely associated with its functional performance. As shown in Figure 1D, CMP solubility increased significantly following ultrasound treatment and exhibited a progressive rise with increasing ultrasound power, indicating enhanced aqueous dispersibility. The increased solubility may therefore be associated with ultrasound-induced conformational rearrangement and changes in protein–water interactions. Acoustic cavitation generated during sonication has been shown to weaken the non-covalent forces that maintain protein conformational stability, potentially leading to a less compact conformation and increased conformational flexibility [25]. This may increase the exposure of hydrophilic groups, enhancing protein hydration and solubility. Wu et al. [26] similarly attributed the improvement in MP solubility to ultrasound-induced conformational adjustment and enhanced protein–water interactions. These findings suggest that ultrasound-induced structural rearrangement contributed to the improved solubility behavior of CMP, which may in turn be associated with its emulsifying performance.

### 2.5. SDS-PAGE Analysis

**Figure 2:**
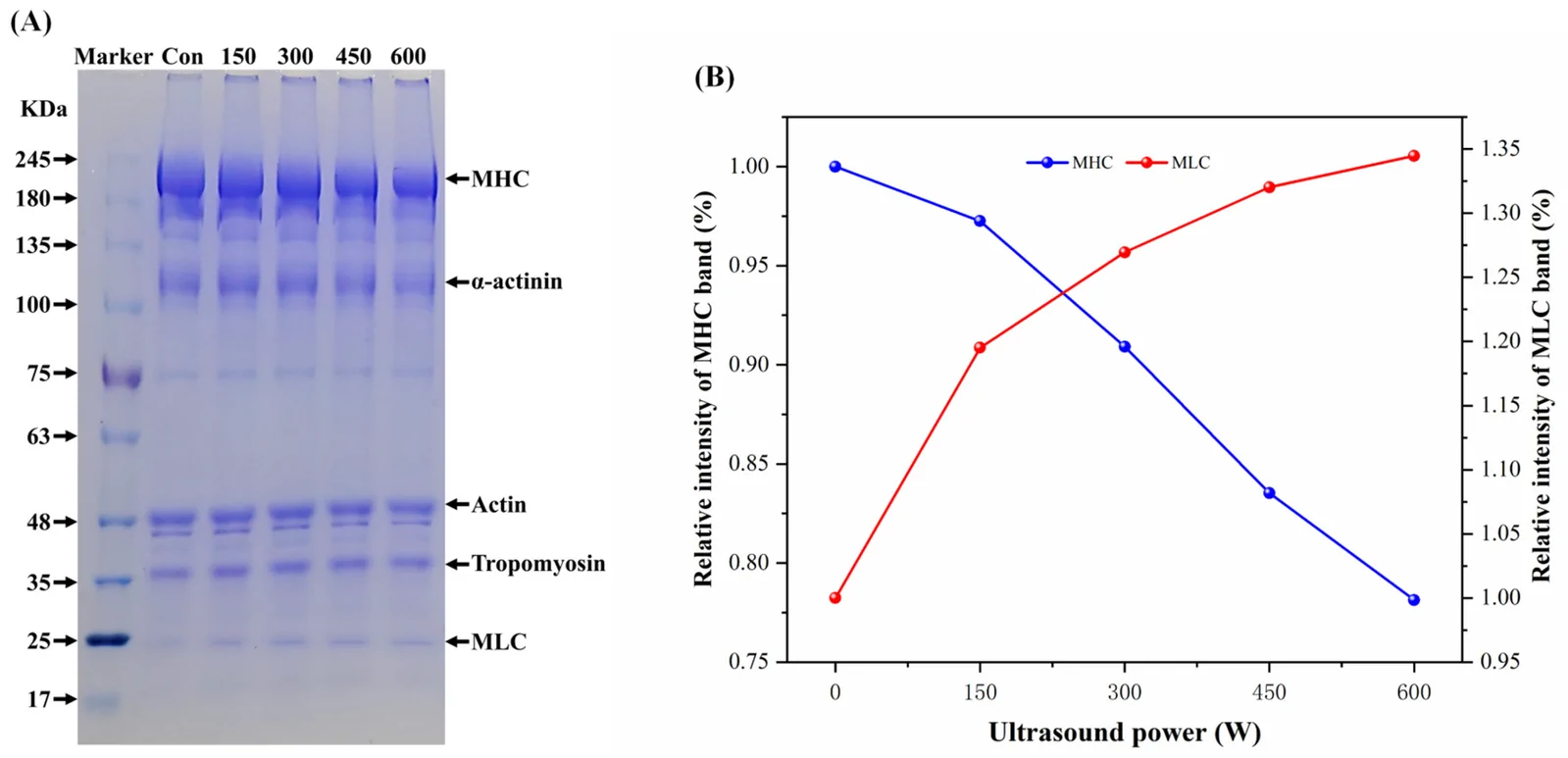
Effects of different ultrasound powers on SDS-PAGE profiles (**A**) and relative band intensities of MHC and MLC (**B**) of CMP.

SDS-PAGE analysis showed that the major protein bands of CMP remained detectable after treatment at different ultrasound powers (Figure 2A). Compared with the control, the positions and numbers of protein bands remained largely unchanged after ultrasound treatment, suggesting that ultrasonic cavitation did not cause substantial degradation or major changes in the detectable protein composition of CMP. However, higher ultrasound power resulted in progressively weaker MHC and actin band intensities, which may reflect changes in the molecular organization or electrophoretic behavior of CMP associated with ultrasound-induced conformational rearrangement [27]. Similar reductions in MHC band intensity following ultrasound treatment have also been reported in fish myofibrillar proteins [3].

Changes in the relative intensity of major protein bands after sonication were further evaluated by measuring the grayscale intensity of SDS-PAGE bands using ImageJ 1.54p (National Institutes of Health, Bethesda, MD, USA), with the quantitative results summarized in Figure 2B. Increasing ultrasound power led to a gradual decline in MHC band intensity, while the relative intensity of the MLC band gradually increased. These changes may reflect alterations in the molecular state or electrophoretic behavior of the corresponding protein components following ultrasound treatment, consistent with previous observations in ultrasound-treated myofibrillar proteins [28].

### 2.6. Secondary Structure Analysis

**Figure 3:**
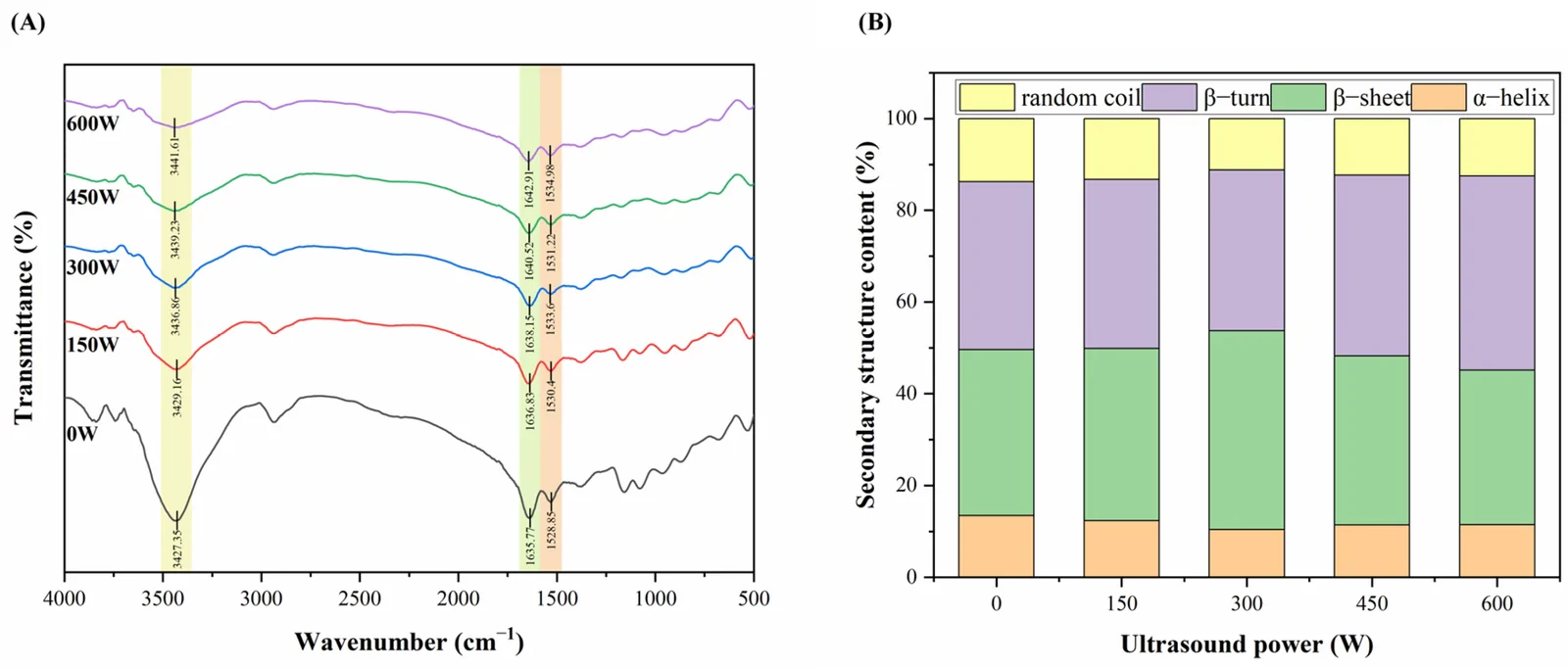
Effects of different ultrasound powers on FTIR spectra (**A**) and secondary structure content (**B**) of CMP.

Secondary-structure variations in CMP were further characterized by FTIR spectroscopy. The FTIR spectra in Figure 3A showed three characteristic peaks, which were sequentially assigned to the amide A, amide I, and amide II bands from left to right. The amide A band (3600–3000 cm^−1^) corresponds mainly to N-H stretching vibrations. In the amide I region (1600–1700 cm^−1^), the spectral signal mainly originated from C=O stretching, whereas the amide II region (1500–1600 cm^−1^) was primarily associated with coupled C-N stretching and N-H bending vibrations. Because the amide I band is highly responsive to secondary-structure changes in proteins, it is commonly used for conformational analysis [29]. In the amide A region, the peak gradually shifted from 3427 to 3441 cm^−1^ as ultrasound power increased, together with slight broadening of the band. This spectral displacement may reflect modification of the local hydrogen-bonding environment in CMP, suggesting changes in N-H-associated hydrogen bonding. In addition, minor variations in band profiles and signal intensities were observed in the amide I and II regions after ultrasound treatment, indicating conformational adjustment of the protein [29]. Accordingly, the amide I region was further resolved to estimate the relative proportions of different secondary-structure components. The sub-bands corresponding to β-sheet, random coil, α-helix, and β-turn structures were identified at 1600–1640, 1640–1650, 1650–1660, and 1660–1700 cm^−1^, respectively [30].

Secondary-structure composition of CMP was estimated from the deconvoluted and curve-fitted amide I band (Figure 3B). The results showed that in CMP without ultrasound treatment, β-turn (36.62%) and β-sheet (36.19%) were the dominant secondary structures, whereas the α-helix content was 13.43%. As ultrasound power increased, the α-helix proportion declined from 13.43% to 11.48%, along with a slight decrease in random coil content. In contrast, β-turn content rose from 36.62% to 42.34%, while β-sheet content peaked at 43.31% under 300 W treatment before slightly decreasing. The reduction in α-helix content suggests that ultrasound treatment altered the hydrogen-bond interactions associated with the helical structure. These alterations may arise from cavitation-induced microdomain effects, including localized thermal accumulation, transient compression, and intense shear forces. Under these conditions, the intramolecular hydrogen-bonding network of CMP may be affected, contributing to a shift in protein conformation [31]. Moderate ultrasound treatment further promoted secondary-structure rearrangement in CMP, as reflected by a reduced α-helix proportion, increased β-turn content, and altered β-sheet distribution, indicating protein conformational changes [32]. Meanwhile, the elevated β-sheet proportion may reflect changes in intermolecular interactions between protein molecules [33]. Overall, ultrasound treatment altered the secondary-structure composition of CMP, with these conformational changes potentially contributing to the observed increases in surface hydrophobicity and solubility, as well as enhanced emulsifying performance.

### 2.7. Emulsification Properties

#### 2.7.1. EAI and ESI

**Figure 4:**
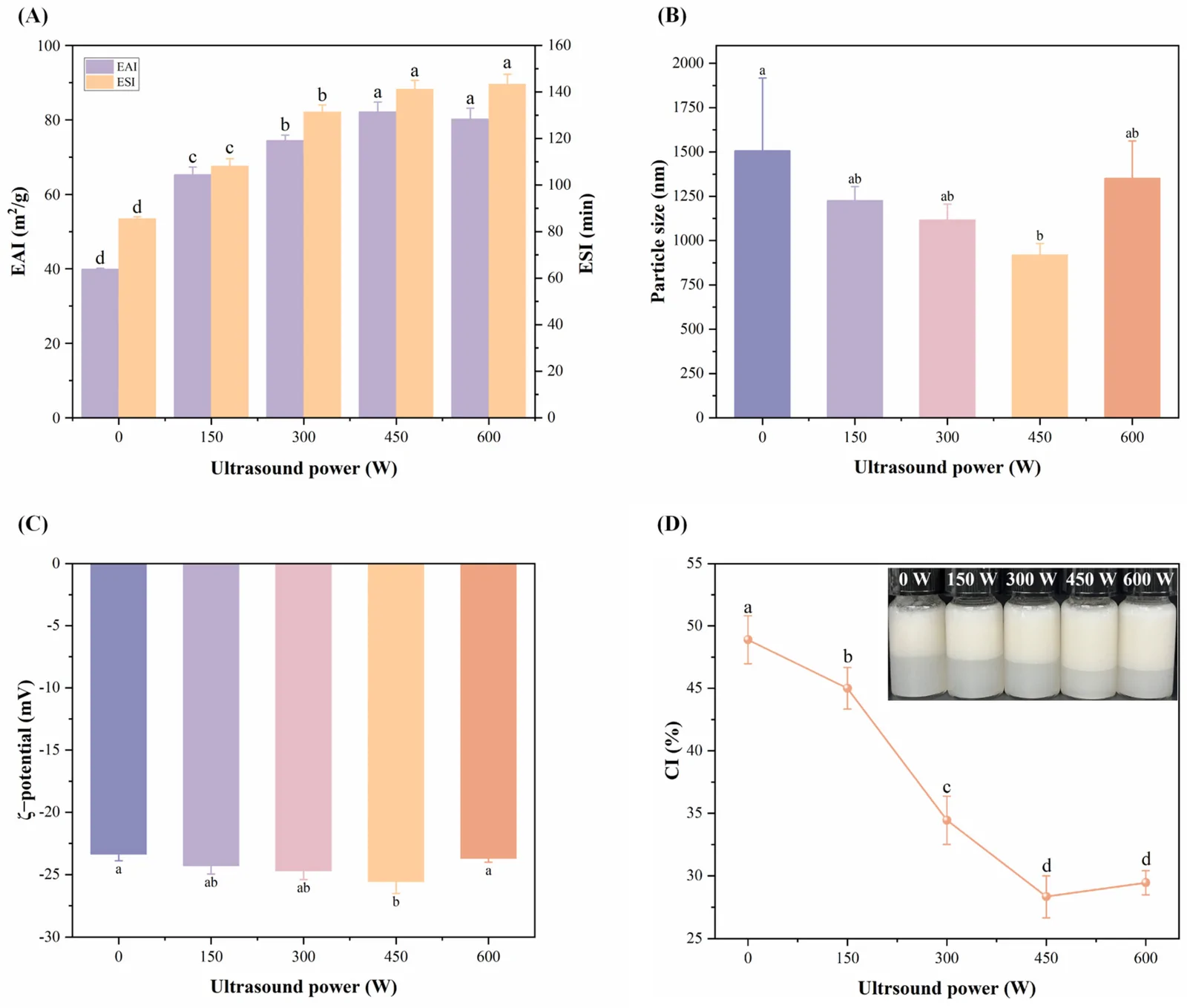
Effects of different ultrasound powers on EAI and ESI (**A**), particle size (**B**), ζ−potential (**C**), and CI (**D**) of CMP−stabilized emulsions. For each parameter, lowercase letters indicate significant differences among treatments (*p* < 0.05).

EAI and ESI provide information on protein behavior during emulsion formation and stabilization. EAI mainly reflects protein interfacial adsorption efficiency, whereas ESI characterizes emulsion resistance to destabilization during storage. The EAI of CMP increased markedly from 39.93 to 82.17 m^2^/g (Figure 4A), as the ultrasound power increased from 0 to 450 W. This improvement may be related to the increased surface hydrophobicity and solubility of CMP (Figure 1C,D), which may reflect ultrasound-induced conformational rearrangement and improved molecular dispersibility, thereby potentially facilitating interactions between CMP and the oil–water interface [34]. A similar relationship between conformational unfolding and emulsifying activity has been reported for duck liver protein treated with ultra-high pressure and stabilizers [35]. Under the conditions used in this study, 450 W may have provided a favorable balance between improved molecular dispersibility and increased exposure of hydrophobic groups, contributing to the highest EAI observed. By contrast, the slight reduction in EAI at 600 W may be associated with further structural modification of CMP. At this treatment intensity, further conformational rearrangement and possible intermolecular interactions may partially reduce the accessibility of exposed hydrophobic residues, potentially explaining the lower surface hydrophobicity observed at 600 W (Figure 1C). This may reduce the availability of interfacially active sites, thereby limiting protein migration toward the oil–water interface [33]. ESI increased with increasing ultrasound power over the tested range (Figure 4A). Zhou et al. [14] attributed the improved emulsion stability under suitable ultrasound conditions to increased accessibility of hydrophobic domains, which may favor protein association at the oil–water boundary and contribute to the formation of a more effective protective interfacial layer. Overall, ultrasound treatment improved the EAI and ESI of CMP compared with the untreated sample under the conditions tested.

#### 2.7.2. Particle Size and ζ-Potential

Emulsion stability is strongly influenced by particle size characteristics. As ultrasound power increased to 450 W, the hydrodynamic particle size of CMP-stabilized emulsions gradually decreased, reaching the lowest value at this treatment level (Figure 4B). The formation of smaller particles may be related to the improved dispersibility of CMP after ultrasound treatment, which may have facilitated droplet breakup during emulsification. However, at 600 W, the particle size increased slightly but remained significantly smaller than that of the control. This increase may be associated with further ultrasound-induced conformational modification of CMP, which could affect its emulsifying behavior and result in less favorable droplet dispersion. Consistent with this interpretation, the changes in sulfhydryl-related indicators and surface hydrophobicity suggest that high-power ultrasound further altered the structural state of CMP, which may have affected its emulsifying performance and consequently the droplet-size characteristics of the emulsions [33,36].

In addition, ζ-potential is commonly used to assess droplet charge and electrostatic stability in emulsion systems. Figure 4C shows that all emulsions exhibited negative ζ-potential values. The ζ-potential became progressively more negative as ultrasound power increased to 450 W, reaching −25.57 mV, but its magnitude decreased at 600 W. A similar pattern was reported for ultrasound-treated pork MPs by Liu et al. [28]. Ultrasonic treatment may induce partial unfolding of CMP, allowing internal charged groups to become more accessible and potentially affecting electrostatic repulsion among emulsion droplets [37]. The decrease in ζ-potential magnitude at 600 W showed a trend consistent with the variation observed in droplet size. Excessive sonication may have induced further conformational modification of CMP, which could affect the surface charge characteristics of the emulsion droplets and potentially influence electrostatic repulsion between droplets [28].

#### 2.7.3. Creaming Index

The creaming index (CI) is commonly applied to evaluate emulsion storage stability, as it reflects the degree of droplet aggregation and gravity-driven phase separation within the system [3]. A lower CI value indicates better storage stability. As presented in Figure 4D, ultrasound treatment altered the CI values of CMP emulsions. Among all groups, the control emulsion exhibited the highest CI value, indicating the poorest storage stability under the tested conditions. In contrast, ultrasound treatment markedly reduced the CI, with the lowest value observed at 450 W, suggesting the highest emulsion stability among the tested treatments. The improved stability after moderate ultrasound treatment may be associated with smaller droplets (Figure 4B) and greater ζ-potential magnitude (Figure 4C), as these changes favored droplet dispersion and reduced the tendency for aggregation and phase separation. Furthermore, Zhu et al. [38] suggested that appropriate ultrasound treatment reinforced the interfacial layer, thereby limiting droplet mobility and improving emulsion stability.

### 2.8. Microstructural Observation

#### 2.8.1. Optical Microscopy

**Figure 5:**
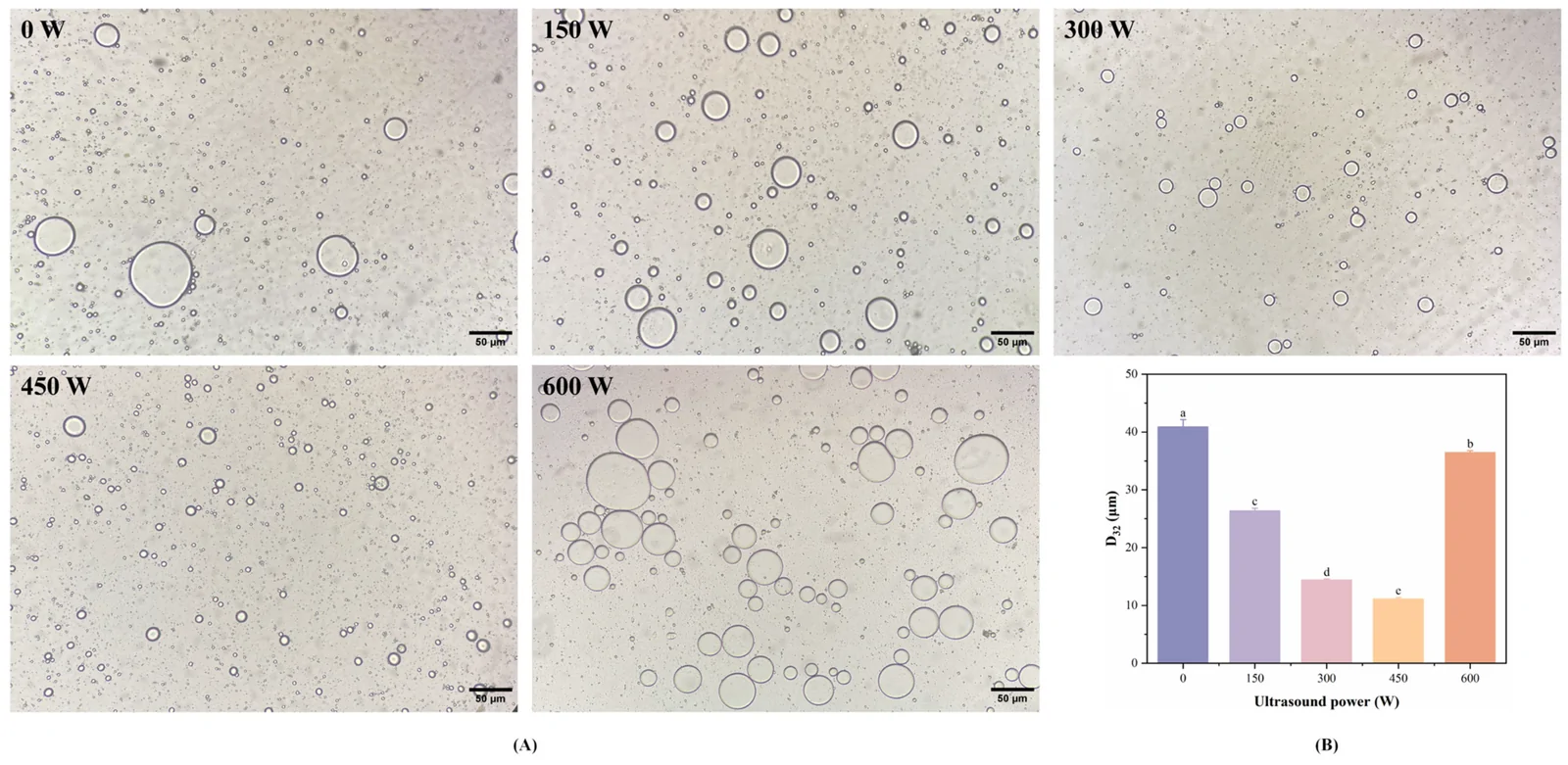
(**A**) Optical micrographs and (**B**) Sauter mean diameter (*D*_32_) of CMP-stabilized emulsions prepared using CMP solutions subjected to different ultrasound treatment powers. Scale bar = 50 μm. Different lowercase letters indicate significant differences among treatments (*p* < 0.05).

Micrographs of emulsions prepared using ultrasound-treated CMP solutions are shown in Figure 5A. The untreated emulsion exhibited large droplets with a highly heterogeneous distribution. After 150 W treatment, droplets became slightly smaller, but the overall distribution remained uneven. Under 450 W ultrasound, the emulsion showed markedly smaller droplets with a more uniform and compact arrangement, clear boundaries, and no obvious aggregation. In contrast, the 600 W treatment resulted in enlarged droplets, poorer distribution uniformity, and evident aggregation. The Sauter mean diameter (*D*_32_) was calculated from the area-equivalent diameters of individual droplets in the microscopic images (Figure 5B). This image-derived mean differs in definition and weighting from the DLS-derived hydrodynamic particle size. The *D*_32_ value decreased from 40.91 μm for the untreated emulsion to 11.12 μm after 450 W ultrasound treatment, whereas further increasing the ultrasound power to 600 W resulted in an increased *D*_32_ value of 36.50 μm. These results were consistent with the microscopic observations. These observations indicate that moderate ultrasound exposure noticeably improved the microstructure of CMP-stabilized emulsions. Ultrasound-generated mechanical forces and cavitation may have contributed to the disruption of protein aggregates and enhanced protein dispersion, possibly favoring droplet size reduction during emulsification [39]. In addition, ultrasound-induced conformational changes and increased surface hydrophobicity may improve the emulsifying ability of CMP, thereby contributing to the formation of smaller and more stable droplets. Wang et al. [40] reported a similar power-dependent trend in rice bran protein–tannic acid emulsions, with the 450 W treatment group exhibiting the smallest and most uniformly distributed droplets, whereas higher-power treatment was associated with larger droplets and greater aggregation.

#### 2.8.2. SEM

**Figure 6:**
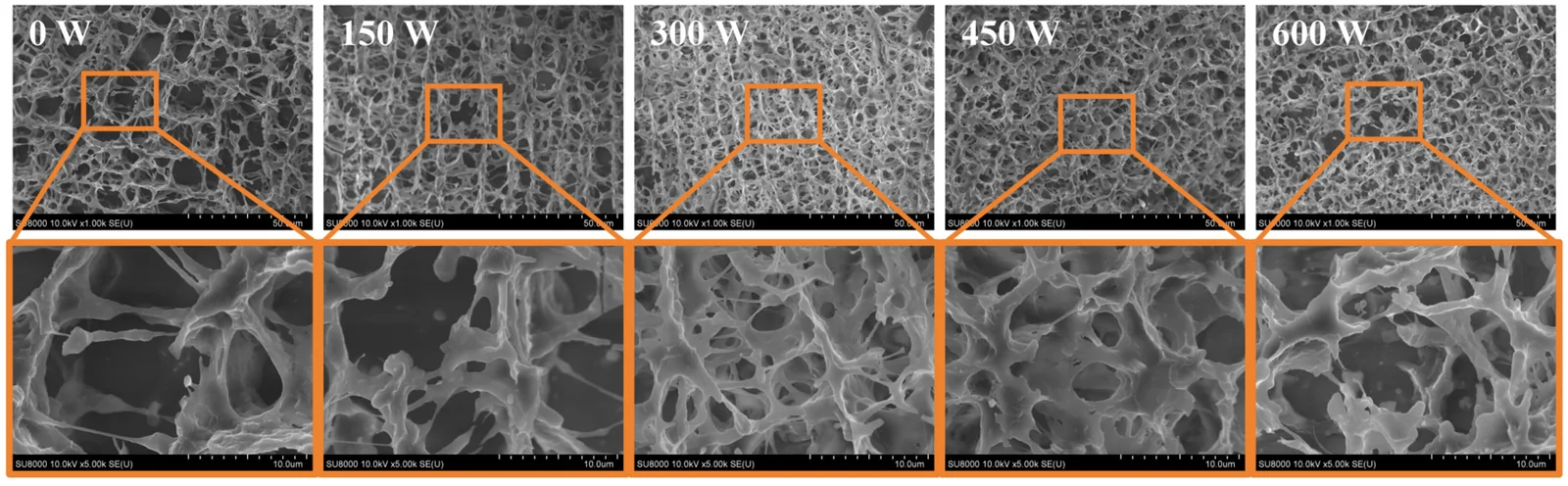
SEM micrographs of CMP gels prepared from solutions treated at different ultrasound power levels.

The microstructure of CMP gels prepared from ultrasound-treated CMP solutions was examined by SEM. In the untreated sample, the network structure appeared relatively loose and fragmented, with irregularly distributed large pores (Figure 6). After ultrasound treatment, the CMP gel network gradually became more uniform and compact, with reduced pore dimensions, particularly under 300 W and 450 W treatments. Wang et al. [41] similarly suggested that moderate sonication could promote changes in MP molecular association and conformation, resulting in a denser gel matrix with smaller pores. These structural changes may be related to ultrasound-induced conformational rearrangement and enhanced intermolecular interactions, thereby contributing to the development of a more ordered and compact network [18]. However, at 600 W, some regions exhibited thinner network walls, enlarged pores, and structural irregularities. This behavior may stem from oxidative modification and protein re-association under excessive ultrasound exposure, potentially compromising network stability. The gel microstructure provides complementary insight into the network-forming behavior of ultrasound-treated CMP, and the similar trends in gel morphology and emulsion properties may reflect their shared dependence on changes in protein conformation and association.

### 2.9. Rheological Properties

**Figure 7:**
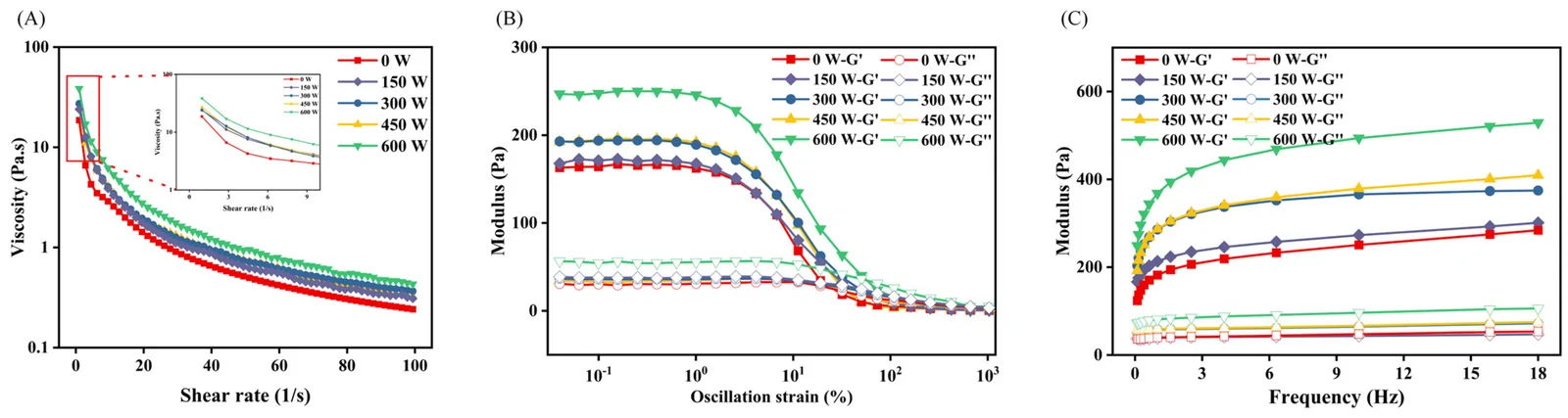
Rheological properties of CMP-stabilized emulsions prepared under different ultrasound power levels: viscosity (**A**); storage modulus (G′) and loss modulus (G″) during strain sweep (**B**); G′ and G″ during frequency sweep (**C**).

The flow curves in Figure 7A reveal a pronounced decline in apparent viscosity for all CMP emulsions throughout the applied shear-rate interval, suggesting shear-thinning behavior and non-Newtonian flow characteristics. This phenomenon may primarily be associated with the breakdown of droplet aggregates and shear-induced alignment of dispersed droplets, which may subsequently reduce viscosity [42]. At the same shear rate, emulsions prepared using ultrasound-treated CMP solutions showed markedly higher viscosity than the untreated control (0 W), with an overall increase observed at higher ultrasound powers. This result may be explained by conformational changes induced by sonication. Such changes could increase the accessibility of hydrophobic domains, which may facilitate protein interactions with oil droplets and contribute to the development of a compact interfacial layer [36]. In addition, the smaller droplet size observed in emulsions prepared with ultrasound-treated CMP may also have contributed to the increased apparent viscosity.

The elastic component of the emulsion system, characterized by the storage modulus (G′), reflects solid-like behavior, while the loss modulus (G″) describes the viscous contribution associated with flow characteristics. The balance between these two moduli provides insight into the viscoelastic properties of emulsions. Figure 7B shows the variations in G′ and G″ of emulsions prepared using CMP solutions treated at different ultrasound power levels. Within the low-strain region, G′ remained higher than G″ across all treatment groups, suggesting that the emulsion systems were predominantly elastic, which is characteristic of gel-like viscoelastic behavior. Compared with the control, ultrasound treatment markedly elevated the storage and loss moduli, especially at medium and high power levels, indicating a stronger elastic response and suggesting a more developed emulsion network [36].

The dynamic mechanical response of the internal structure in the emulsion samples was further examined by frequency sweep measurements. Figure 7C shows that G′ and G″ increased progressively with frequency in all emulsions. Throughout the tested frequency range, G′ remained above G″, indicating that the systems were dominated by elastic characteristics. A similar rheological pattern was described by Rajasekaran et al. [8]. The gradual rise in G′ further suggests the development of a more interconnected emulsion network. Notably, emulsions prepared with ultrasound-treated CMP showed substantially higher G′ and G″ values than the control, with both moduli generally rising with ultrasound power. These findings indicate that ultrasound treatment strengthened the internal network architecture and improved the viscoelastic performance of the emulsions [40]. Li et al. [36] similarly linked high-intensity sonication to enhanced rheological behavior and greater stability of MP emulsions. Nevertheless, the overall emulsion stability depended on multiple properties, and higher viscoelastic moduli did not necessarily indicate better overall stability.

## 3. Materials and Methods

### 3.1. Materials

*C. ectenes taihuensis* specimens were collected from Taihu Lake, China. The fresh fish were maintained under refrigerated conditions during transportation and immediately processed upon arrival at the laboratory. The head, viscera, scales, skin, and bones were removed, after which the muscle tissues from approximately 10 fish were collected and pooled for each biological batch. The pooled muscle tissues were minced and stored at −20 °C prior to use. Sunflower oil purchased from a local supermarket served as the oil phase for emulsion preparation. Unless otherwise specified, analytical-grade reagents were obtained from Sinopharm Chemical Reagent Co., Ltd. (Shanghai, China).

### 3.2. Extraction of MP

MP was extracted with minor modifications to the procedure reported by Zhang et al. [43]. For each biological batch, MP was extracted separately from the pooled muscle tissue. Briefly, surimi was combined with an extraction buffer composed of 0.1 M NaCl, 2 mM MgCl_2_, 1 mM EDTA, and 0.1 M Na_2_HPO_4_/NaH_2_PO_4_ (pH 7.0) at a sample-to-buffer ratio of 1:4 (*w*/*v*). The mixture was homogenized at 10,000 rpm for 30 s and subsequently centrifuged at 5000× *g* for 15 min at 4 °C. After removal of the supernatant, the extraction procedure was repeated three times in total to obtain crude MP from *C. ectenes taihuensis*. The crude MP underwent three washing cycles with 0.1 M NaCl solution at a 1:4 (*w*/*v*) sample-to-solution ratio, with the same homogenization and centrifugation conditions applied after each washing step. Before the final centrifugation, the washed precipitate was suspended in PBS containing 0.6 M NaCl and 20 mM sodium phosphate buffer (pH 7.0), homogenized, and subsequently extracted at 4 °C for 1 h. The extract was filtered through triple-layer gauze and centrifuged again to obtain the MP solution, and its protein concentration was then quantified using the BCA assay (Beijing Lanjieke Technology Co., Ltd., Beijing, China).

### 3.3. Ultrasonic Treatment of MP

MP solution was sonicated using an ultrasonic cell disruptor (SCIENTZ-IID, Ningbo, China) following a slightly modified procedure from Lan et al. [3]. Briefly, a 30 mL portion of MP solution (10 mg/mL) was processed in a 50 mL centrifuge tube. An ice-water bath was maintained throughout sonication to minimize heat accumulation during treatment. The instrument operated at a nominal frequency of 20 kHz, and the 6 mm-diameter titanium probe was positioned 2 cm beneath the liquid surface throughout the treatment. Pulsed sonication was conducted for 15 min at five nominal output power levels (0, 150, 300, 450, and 600 W), with each cycle consisting of 2 s of ultrasound exposure and a 2 s pause. The reported powers refer to the processor’s nominal settings; sample temperature during sonication and the actual acoustic power delivered to the sample were not directly quantified. The treated samples were subsequently maintained at 4 °C until analysis.

### 3.4. Structural Characterization of MP

#### 3.4.1. Total Sulfhydryl (T-SH) Content

The total sulfhydryl content of CMP was determined according to Deng et al. [33], with minor modifications. A reaction buffer was prepared by combining 50 mM PBS (pH 7.0), 8 M urea, 10 mM ethylenediaminetetraacetic acid (EDTA), and 0.6 M NaCl. The CMP solution was adjusted to 2 mg/mL, after which 1 mL was combined with 9 mL of the reaction buffer. The reaction mixture was supplemented with 0.4 mL of 0.1% (*w*/*v*) DTNB solution and incubated at 40 °C for 25 min. A mixture of 50 mM PBS and 0.1% (*w*/*v*) DTNB served as the blank, and sample absorbance was recorded at 412 nm.

#### 3.4.2. Carbonyl Content

Carbonyl groups in CMP were quantified through 2,4-dinitrophenylhydrazine (DNPH) derivatization following the method described by Sun et al. [44]. A 1 mL aliquot of CMP dispersion (4 mg/mL) was combined with an equal volume of DNPH reagent (10 mM prepared in 2 M HCl). The reaction mixture was protected from light and gently agitated every 10 min for 1 h. Next, 200 μL of 20% trichloroacetic acid (TCA) was added to the sample, which was then centrifuged at 10,000× *g* for 5 min to collect the precipitated protein fraction. The pellet underwent three successive washes with ethanol-ethyl acetate (1:1, *v*/*v*), which effectively removed residual DNPH. After washing, 600 μL of 6 M guanidine hydrochloride was added to the pellet, and the resulting suspension was incubated in a water bath at 37 °C for 15 min. After incubation, the suspension was centrifuged at 10,000× *g* for 5 min. A 200 μL portion of the supernatant was subsequently used for absorbance measurement at 370 nm. Carbonyl content was determined from the absorbance using a molar extinction coefficient of ε = 22,000 M^−1^·cm^−1^ and reported as nmol/mg protein.

#### 3.4.3. Surface Hydrophobicity (H_0_)

The surface hydrophobicity of CMP was evaluated by fluorescence spectroscopy using 8-anilino-1-naphthalenesulfonic acid (ANS) as the fluorescent probe [45]. Treated CMP dispersions were adjusted to final concentrations of 0.2–1.0 mg/mL using 20 mM phosphate buffer (pH 7.0). For each concentration, 10 μL of 8 mM ANS solution was introduced into 4 mL of the sample. Fluorescence was measured at 390 nm excitation and 485 nm emission. Surface hydrophobicity was expressed as the initial slope of the linear regression of relative fluorescence intensity against protein concentration.

#### 3.4.4. Solubility

The solubility of CMP was evaluated according to Tao et al. [12]. Briefly, CMP dispersions at 1 mg/mL were subjected to centrifugation (10,000× *g*, 15 min, 4 °C), and the resulting supernatant fraction was retained for analysis. Soluble protein levels were measured with a commercial BCA kit (Beijing Lanjieke Technology Co., Ltd., Beijing, China), and solubility was expressed as follows:

(1) $$\mathrm{Solubility}\;(\%)\;=\;\frac{C_{1}}{C_{0}}\times 100$$

where *C*_0_ and *C*_1_ denote the total protein concentration in the original sample and the protein level in the supernatant after centrifugation, respectively; both are expressed in mg/mL.

#### 3.4.5. Sodium Dodecyl Sulfate–Polyacrylamide Gel Electrophoresis (SDS-PAGE)

The SDS-PAGE procedure was adapted from Zhou et al. [46]. Stacking gels (5%) and separating gels (15%) were prepared. Following centrifugation (10,000× *g* for 5 min), the supernatant was supplemented with loading buffer at a 4:1 (*v*/*v*) ratio and heated at 95 °C for 5 min. The denatured samples (10 μL) were then loaded onto the gel. The voltage was subsequently increased to 120 V and maintained until the tracking dye reached the lower edge of the gel. The gels were subsequently immersed in Coomassie Brilliant Blue R-250 for 30 min and destained until the background became clear. After destaining, the gels were photographed, and ImageJ 1.54p (National Institutes of Health, Bethesda, MD, USA) was used to quantify the intensities of the MHC and MLC bands. After background subtraction, the band intensity of each sample was normalized to that of the corresponding 0 W control, which was set to 1.0.

#### 3.4.6. Fourier Transform Infrared (FTIR) Spectroscopy

FTIR spectra of CMP were collected using a spectrometer (Thermo Fisher Scientific, Madison, WI, USA) according to the method of Zhao et al. [11]. Freeze-dried CMP was mixed thoroughly with KBr at a mass ratio of 1:100 (CMP:KBr, *w*/*w*) and compressed into pellets before measurement. Spectral acquisition was performed at 25 °C over the range of 4000–400 cm^−1^ with 16 scans at a resolution of 4 cm^−1^. After processing with OMNIC 9.0 (Thermo Fisher Scientific, Madison, WI, USA), the spectra were subjected to baseline correction and smoothing, and the amide I region (1600–1700 cm^−1^) was analyzed using PeakFit 4.12 (SeaSolve Software Inc., San Jose, CA, USA) with Gaussian fitting following second-derivative treatment. The relative proportions of β-sheet (1600–1640 cm^−1^), random coil (1640–1650 cm^−1^), α-helix (1650–1660 cm^−1^), and β-turn (1660–1700 cm^−1^) were estimated from the corresponding fitted peak areas, with the fitting quality assessed using R^2^.

### 3.5. Emulsifying Properties

#### 3.5.1. Emulsion Preparation

CMP emulsions were prepared by blending CMP suspension (10 mg/mL) with sunflower oil at a volume ratio of 4:1, followed by two rounds of homogenization at 10,000 rpm for 30 s each.

#### 3.5.2. Emulsifying Activity Index (EAI) and Emulsion Stability Index (ESI)

EAI and ESI of CMP were determined following the procedure described by Wang et al. [18]. Immediately after emulsion preparation, a 50 μL aliquot was collected from the lower portion of the centrifuge tube and mixed with 5 mL of 0.1% (*w*/*v*) SDS solution. The absorbance at 500 nm was recorded as A_0_, and the absorbance was measured again after 10 min to obtain A_10_. EAI and ESI values were subsequently determined according to the equations given below:

(2) $$\mathrm{EAI}\;(m^{2}/g)\;=\frac{2\;\times \;2.303\;\times \;A_{0}\;\times \;DF}{C\;\times \;\varphi \;\times \;10^{4}}$$

(3) $$\mathrm{ESI}\;(\min)=\frac{A_{0}\times 10}{A_{0}-A_{10}}$$

where *A*_0_ and *A*_10_ are the absorbance readings at 0 and 10 min, respectively; *DF* represents the 100-fold dilution factor; *C* indicates the sample protein concentration (10 mg/mL); and *φ* denotes the sunflower oil proportion in the system (20%).

#### 3.5.3. Measurement of Particle Size and ζ-Potential

A Zetasizer Nano ZS system (Malvern Instruments Ltd., Worcestershire, UK) was used to characterize the particle size and ζ-potential of CMP emulsions following the method described by Wang et al. [47]. Before measurement, each emulsion sample was diluted with deionized water to a protein concentration of 0.1 mg/mL and equilibrated at 25 °C for 120 s. Particle size was determined by dynamic light scattering (DLS) based on Brownian diffusion and expressed as hydrodynamic diameter. Particle size and ζ-potential were measured immediately after emulsion preparation. All measurements were performed with three replicates, and the averaged values were subjected to statistical analysis.

#### 3.5.4. Creaming Index (CI)

The CI measurement was determined according to Yu et al. [48], with minor modifications. Fresh emulsions were placed in glass vials and stored at 4 °C for 14 days. The CI was then computed with the equation shown below:

(4) $$\mathrm{CI}\;(\%)\;=\;\frac{H_{C}}{H_{E}}\times 100$$

where *H_C_* refers to the bottom transparent layer height (cm), while *H_E_* represents the overall emulsion height (cm).

#### 3.5.5. Optical Microscopy

CMP-stabilized emulsions were observed using the bright-field imaging mode of a fluorescence microscope (Ti, Nikon, Tokyo, Japan), according to Rajasekaran et al. [8]. A small portion of each emulsion was transferred onto a clean glass slide, followed by microscopic observation at 10× magnification and image acquisition. The droplet size was analyzed using ImageJ software after calibration with the scale bar. The equivalent diameter of individual droplets was calculated from the measured droplet area, and the Sauter mean diameter (*D*_32_) was determined according to the following equation [49]:

(5) $$D_{32}\;=\;\frac{\sum d_{i}^{3}}{\sum d_{i}^{2}}$$

where *d_i_* represents the equivalent diameter of individual droplets.

### 3.6. Scanning Electron Microscopy (SEM)

The surface morphology of CMP gels subjected to different ultrasound power treatments was examined using an SU8010 scanning electron microscope (HITACHI, Tokyo, Japan) according to the modified method of Senphan et al. [50]. CMP gels were prepared using a two-stage heating procedure in a water bath at 40 °C for 30 min, followed by 90 °C for 30 min. The samples were then cooled in an ice-water bath for 30 min and stored at 4 °C overnight. Briefly, samples were immersed in 2.5% glutaraldehyde prepared in 0.2 M phosphate buffer (pH 7.2) and fixed for 3 h. The fixed samples were subsequently dehydrated through ethanol solutions of 30%, 50%, 70%, 80%, 90%, and 100% (*v*/*v*). Before SEM imaging, the lyophilized specimens were affixed to conductive adhesive tape and subsequently gold-sputter coated under vacuum. Surface morphology was examined at 10 kV under magnifications of 1000× and 5000×.

### 3.7. Rheological Measurements

The rheological behavior of CMP emulsions was analyzed using a DHR-2 rheometer (TA Instruments, New Castle, DE, USA) fitted with a 40-mm parallel-plate system. All measurements were performed at 25 °C with a fixed plate gap of 1 mm. Flow curves were generated under steady shear by varying the shear rate from 0.1 to 100 s^−1^, while apparent viscosity was recorded simultaneously. For oscillatory rheological measurements, a strain sweep from 0.01% to 1000% was first conducted at 1 Hz to determine the linear viscoelastic region (LVR). Subsequently, frequency sweeps were performed from 0.1 to 18 Hz within the determined LVR, with the storage modulus (G′) and loss modulus (G″) recorded throughout the test [8].

### 3.8. Data Analysis

Data are expressed as mean ± standard deviation (SD) from three independently sampled and prepared MP batches. Statistical analysis was performed in IBM SPSS Statistics 26 (IBM Corp., Armonk, NY, USA) using one-way ANOVA and Tukey’s post hoc test for pairwise comparisons. The assumption of homogeneity of variance was checked using Levene’s test. Differences were considered significant at *p* < 0.05. Graphical representations were prepared using Origin 2022 (OriginLab Corporation, Northampton, MA, USA).

## 4. Conclusions

The present study shows that ultrasound power significantly affected the structural characteristics of CMP and its ability to stabilize emulsions. Ultrasound-induced structural changes in CMP, as evidenced by a lower α-helix proportion, increased β-turn content, higher surface hydrophobicity, elevated carbonyl content, and reduced total sulfhydryl content. These changes suggest conformational rearrangement accompanied by oxidation-related modification. Moderate ultrasound further improved CMP solubility, EAI, and ESI, indicating improved emulsion stabilization performance. The corresponding emulsions contained finer and more uniformly distributed droplets, greater ζ-potential magnitude, and lower creaming index values, indicating strengthened electrostatic repulsion and effective inhibition of droplet aggregation. SEM analysis revealed a more homogeneous and compact CMP gel structure after moderate ultrasound treatment, while rheological analysis indicated enhanced viscoelastic properties of the emulsions. SDS-PAGE analysis indicated no marked changes in the electrophoretic profiles of CMP subunits after ultrasound treatment. Overall, ultrasound-driven conformational rearrangement was associated with enhanced interfacial stabilization capability and emulsion stability of CMP under the tested conditions. These findings suggest that appropriate ultrasound treatment may improve the functionality of CMP as a protein-based emulsifying ingredient in freshwater fish protein-based emulsion systems. Further investigation under different formulation and storage conditions is needed to assess the broader applicability of ultrasound-treated CMP, including its performance in real food matrices and the scalability and processing feasibility of ultrasound treatment. Additionally, future studies could explore the integration of ultrasound-treated CMP with ionic liquid/deep eutectic solvent-based intelligent freshness-monitoring systems for high-protein aquatic foods [51].

## Data Availability

The original data presented in the study are openly available in https://www.scidb.cn/en/s/rIRbAr (accessed on 24 September 2026).
